# An age-independent gene signature for monitoring acute rejection in kidney transplantation

**DOI:** 10.7150/thno.42110

**Published:** 2020-05-25

**Authors:** Brian I Shaw, Daniel K. Cheng, Chaitanya R. Acharya, Robert B Ettenger, Herbert Kim Lyerly, Qing Cheng, Allan D Kirk, Eileen T Chambers

**Affiliations:** 1 Department of Surgery, Duke University Medical Center, Durham, United States; 2 Department of Pediatrics, Duke University Medical Center, Durham, United States; 3 Department of Pediatrics, UCLA Mattel Children's Hospital, Los Angeles, United States

**Keywords:** kidney transplant, acute rejection, gene expression, pediatrics, big data

## Abstract

Acute rejection (AR) remains a significant problem that negatively impacts long-term renal allograft survival. Numerous therapies are used to prevent AR that differ by center and recipient age. This variability confounds diagnostic methods.

**Methods**: To develop an age-independent gene signature for AR effective across a broad array of immunosuppressive regimens, we compiled kidney transplant biopsy (n=1091) and peripheral blood (n=392) gene expression profiles from 12 independent public datasets. After removing genes differentially expressed in pediatric and adult patients, we compared gene expression profiles from biopsy and peripheral blood samples of patients with AR to those who were stable (STA), using Mann-Whitney U Tests with validation in independent testing datasets. We confirmed this signature in pediatric and adult patients (42 AR and 47 STA) from our institutional biorepository.

**Results**: We identified a novel age-independent gene network that identified AR from both kidney and blood samples. We developed a 90-probe set signature targeting 76 genes that differentiated AR from STA and found an 8 gene subset (DIP2C, ENOSF1, FBXO21, KCTD6, PDXDC1, REXO2, HLA-E, and RAB31) that was associated with AR.

**Conclusion**: We used publicly available datasets to create a gene signature of AR that identified AR irrespective of immunosuppression regimen or recipient age. This study highlights a novel model to screen and validate biomarkers across multiple treatment regimens.

## Introduction

Despite advancements in clinical care for kidney transplant patients, long term outcomes remain sub-optimal 1-3. The reported incidence of acute rejection (AR)—including antibody mediated rejection (ABMR) and T cell mediated rejection (TCMR)—in the first year after transplantation varies depending on the immunosuppression utilized. It is typically higher with steroid and calcineurin inhibitor minimization or Belatacept-based regimens, though these regimens are often preferred for younger recipients as the reduction in long-term side effects is thought to offset the increased risk of early, treatable AR 4-6. Regardless, AR has been associated with decreased long-term allograft survival in both pediatric and adult studies 7-9. Additionally, TCMR has been correlated with formation of *de novo* donor specific antibody (*dn*DSA) 10 which is strongly associated with premature allograft loss 11. Finally, AR is often associated with inflammation within areas of interstitial fibrosis and tubal atrophy (i-IFTA) 12 at one year that is also correlated with decreased allograft survival 13. Immune monitoring to detect AR allows for early intervention and decreased graft damage, but diagnostic methods, particularly those relying on molecular signatures, are likely confounded by differences in the immunosuppressive strategies used, and these differences are non-uniformly distributed by recipient age.

Recently, immune monitoring has focused on the development of gene signatures for AR in kidney transplantation derived from both renal parenchymal samples and from peripheral blood. Examination of the transcriptome from renal parenchymal tissue has more fully characterized events occurring within the kidney in order to subclassify acute rejection events and help adjudicate difficult to interpret biopsies 14-17. In a related but distinct way, attempts to create a peripheral gene expression signature of AR have also progressed to obviate the need for allograft biopsy and improve the logistics of graft surveillance 18-21. Recent multi-center studies have utilized a peripheral blood signature to discriminate between stable grafts and those undergoing AR 22.

Interestingly, the combination of data from renal parenchymal and peripheral blood signatures to define a more complete signature of AR has been infrequently pursued 23-25. Although there are likely differences in gene expression between the two compartments, local events often mediate systemic changes. Moreover, the non-invasive nature of a peripheral test is clinically more attractive, given complications associated with percutaneous biopsy 26. Additionally, most studies separate adult and pediatric patients, meaning that signatures may not be broadly applicable. Many of these prior studies were performed on a common microarray platform and have all been uploaded into the publicly accessible Gene Expression Omnibus (GEO) 27. Given this wealth of information and the opportunity to combine datasets, we aimed to create a new peripheral signature of AR that would be able to detect both TCMR and ABMR in pediatric and adult patients, regardless of immunosuppression regimen utilized.

## Materials and Methods

### Human genomic data collection

A total of 1091 renal gene expression profiles were collected from 7 independent NCBI Gene Expression Omnibus datasets: GSE21374, GSE22459, GSE36059, GSE50058, GSE7392, GSE9493, and GSE25902 (pediatric) 13,15,18,28-31. In addition, we obtained 392 gene expression profiles of peripheral blood cells derived from 5 GEO datasets: GSE14346, GSE15296, GSE24223, GSE46474, and GSE20300 (pediatric) 21,32-35. Complementing the raw expression data, we also obtained clinical data from a subset of the samples with AR, including both ABMR and TCMR, stable (STA), borderline rejection, chronic allograft nephropathy (CAN), and interstitial fibrosis/tubular atrophy (IF/TA).

### Normalization of gene expression data

Gene expression profiles of all datasets were measured using Affymetrix U133A or U133 Plus 2.0 expression array. Each dataset selected for this study contained clinical outcome data and patients' unique IDs were also collected from series matrix files (GEO) to ensure there was no redundancy in the sample set. Raw Affymetrix expression CEL files from each dataset were robust multi-array average normalized independently using Expression Console Version 1.1 (Affymetrix, Santa Clara, CA). All data were filtered to include those probes on the HG-U133A platform. Batch effects were mitigated using surrogate variable analysis (SVA) 36.

### Selection and analysis of institutional cohort

To further develop a gene signature of early AR, a total of 89 pediatric and adult patients age 1 to 78 transplanted between July 2009 to July 2017 were selected from our institutional biorepository. They were characterized as AR (39 TCMR, 1 ABMR, and 2 Borderline)—with samples within the 30 days preceding the rejection event—or STA without rejection during the first year after transplantation. Immunosuppression protocols included induction with basiliximab, daclizumab, or rabbit anti-thymocyte globulin, while maintenance regimens included use of tacrolimus, cyclosporine, azathioprine, belatacept, sirolimus, and/or mycophenolate mofetil with or without steroids, including some patients on full steroid withdrawal regimens. Cryopreserved peripheral blood mononuclear cell mRNA expression of genes identified in our microarray data was measured using Applied Biosystems™ TaqMan™ Array Cards and Plates (Thermo Fisher, Waltham, MA). All samples were collected from patients with informed consent and all related procedures were performed with the approval of the Duke Institutional Review Board (Pro00093938).

### Statistics analyses

Mann-Whitney U Tests were used to identify genes that were differentially expressed between AR and STA groups. We also used a multivariable Cox-regression survival analysis for risk of AR (with the multiple variables being different gene expression values) to identify genes associated with freedom from AR. Shotgun Stochastic Search in Regression (SSS) was used for assigning coefficients to genes that were identified in our previous step 37. Receiver operating characteristic (ROC) curves were used to assess the diagnostic ability of our signatures in a binary classification system. Gene set enrichment analysis was performed using *Enrichr* 38. A gene network was created using *STRING v11* 39, *Reactome* pathway analysis and *GO* Biological Process analysis were also completed 40-42. To assess if the expression of selected 8 genes was truly an independent risk factor of AR, we performed a multivariable logistic regression analysis using generalized linear models (glm) including the clinical variables of race, gender, age, and treatment (use of depletional induction, and/or use of belatacept based maintenance immunosuppression) with a p<0.05 considered significant. Statistical analyses were performed using Prism 6 (GraphPad, San Diego, CA), Matlab 2014a (Mathworks, Natick, MA), R 3.4.0 (Project for Statistical Computing Vienna, Austria), STATA 15 (STATA Corp, College Station, TX) or STATISTICA 7 (Dell, Round Rock, Tx).

## Results

### Sample normalization

**Figure 1:**
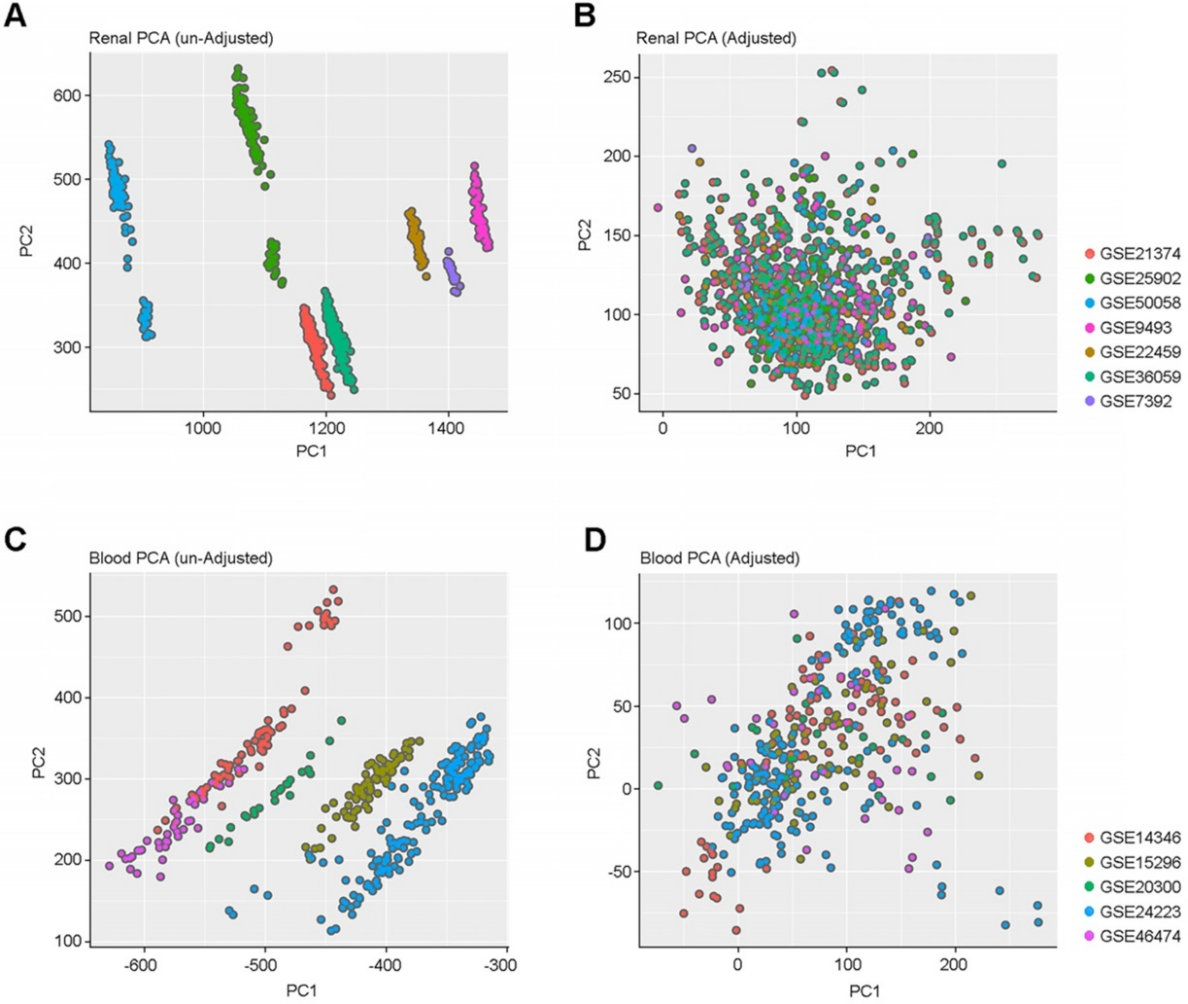
** PCA Plots of batch effect normalization. (A & C)** PCA plots before and after normalization among renal samples**. (B & D)** PCA plots before and after normalization among blood cell samples. These plots show the gene expression profiles of the samples plotted on the first two principal components. Each point represents a sample, and samples from the same data set have the same color. We demonstrate that there are no batch effects.

To capture the heterogeneity of renal allograft rejection, we compiled a large collection of gene expression profile data from either kidney allograft parenchymal biopsy specimens (n=1091) or peripheral blood (n=392) obtained from 12 independent public datasets. Allograft and peripheral blood gene expression profiles showed expression differences among samples obtained from different data sets (**Figure 1A and 1C**). All the gene expression data were combined and batch effects in the combined data were corrected using SVA. (**Figure 1B and 1D**)**.**

### Gene expression differences between adult and pediatric samples

**Figure 2:**
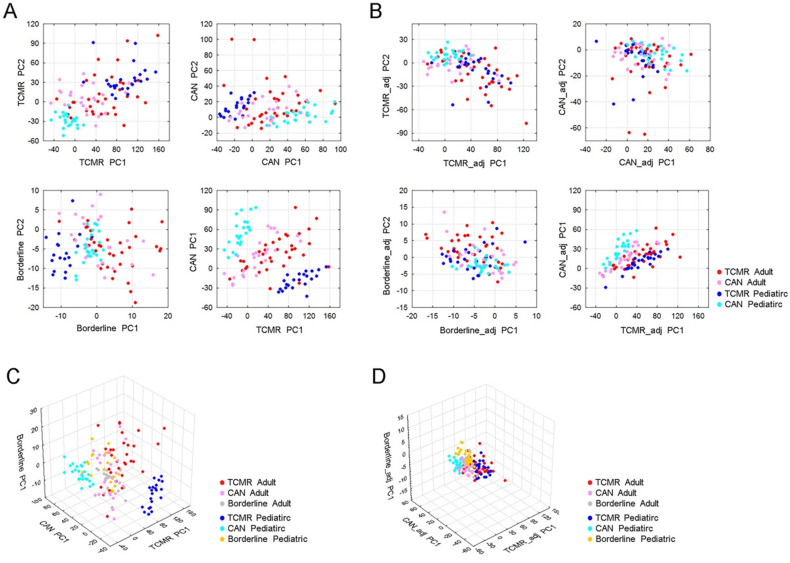
** Discordant gene expression profiles between adult and pediatric cases with renal allograft rejection. (A)** PCA of TCMR, CAN and borderline rejection associated genes reveal significant differences in TCMR and CAN gene profiles between adult and pediatric patients, but not in borderline samples. The upper left panel shows PCA of TCMR using first two principle components (PC1 and PC2) of differentially expressed probe sets between TCMR and STA. The bottom left panel shows PCA of borderline samples using first two principle components (PC1 and PC2) of differential expressed probe sets between borderline and STA. The upper right panel shows PCA of CAN using the first two principle components (PC1 and PC2) of differentially expressed probe sets between CAN and STA. The bottom right panel shows sample distribution defined using PC1 of TCMR associated probe sets and PC1 of CAN associated probe sets, colored by sample type. **(B)** PCA of TCMR, CAN, and borderline rejection after removal of Age-related differentially expressed genes. **(C)** 3D PCA of TCMR, CAN and borderline associated genes prior to removing differentially expressed genes between children and adults. **(D)** 3D PCA of TCMR, CAN and borderline associated genes after removing differentially expressed genes between children and adults yields.

Using all patients (pediatric or adult) we utilized 45,782, probe sets to define expression levels in one of three clinical phenotypes—T Cell Mediated Rejection (TCMR), Borderline rejection, or Chronic Allograft Nephropathy (CAN)—as compared to STA patients (p<0.001, Mann-Whitney U Test). For each of these phenotypes, we plotted both adult and pediatric samples using the first two principal components of differentially expressed probe sets. We observed that adult and pediatric gene expression was significantly different within the TCMR and CAN clinical groups, but not in the borderline group (**Figure 2A and 2C**).

To define the differences between age groups, we subsequently compared expression profiles between adult and pediatric samples and identified 25,043 probe sets whose expressions in TCMR and/or CAN were significantly different between adult and pediatric samples (p<0.001, Mann-Whitney U Test). After removing these age-group related probe sets, we re-built the principal components of TCMR, CAN and borderline using the remaining of 20,739 probe sets. In doing so, we saw a minimization of differences between adult and pediatric samples within the same histologic subtype, indicated by clustering of the points for adult and pediatric samples of the same histologic type (**Figure 2B and 2D**).

### Identification of gene expression differences in AR

**Figure 3:**
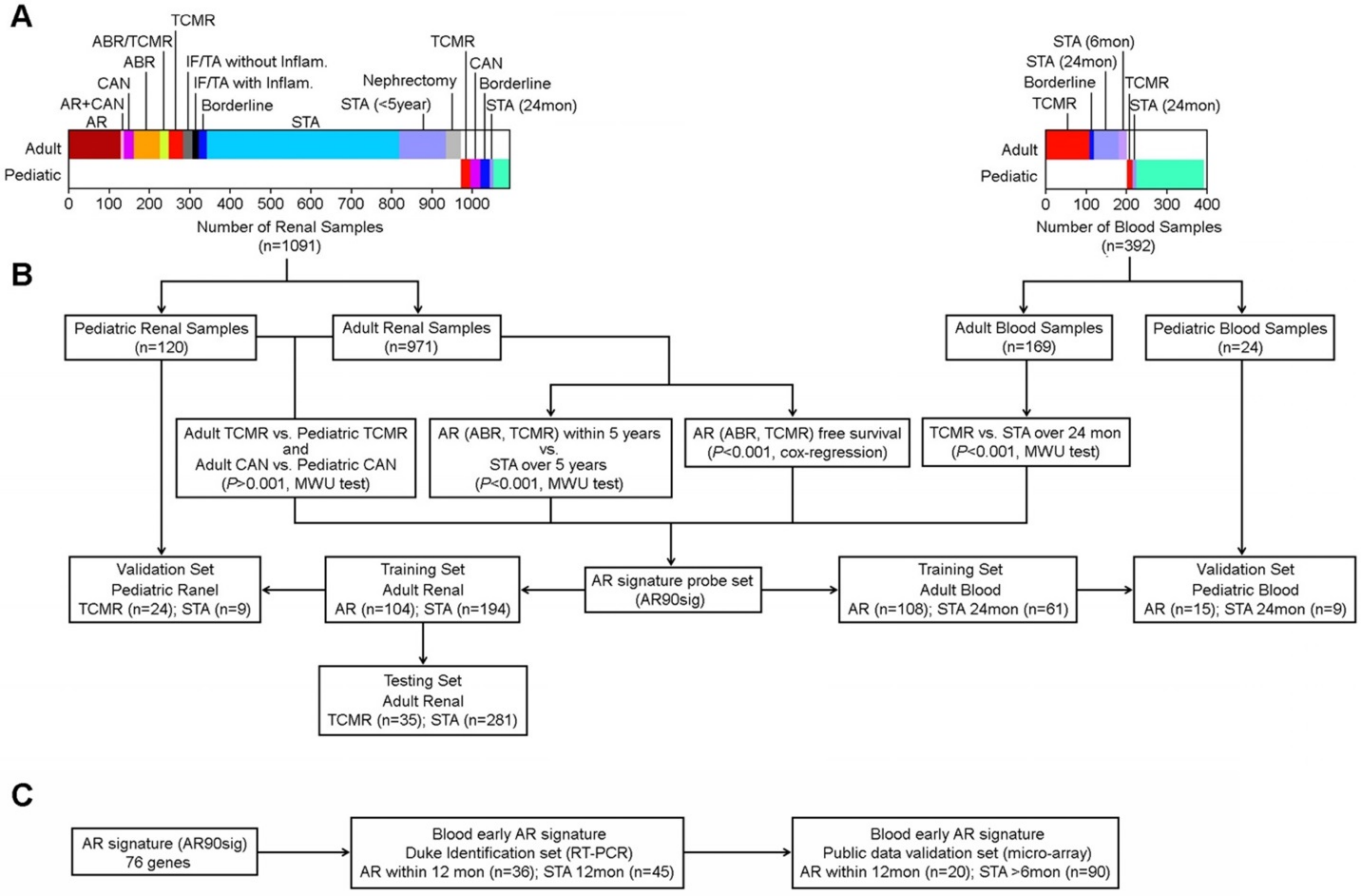
** Workflow for developing age-independent signature of AR using both renal and blood cell samples. (A)** Description of clinical samples used in creation of our initial 90 probe set signature **(B)** Workflow showing the multiple comparisons made to identify our initial 90 probe sets. **(C)** Workflow for identifying early AR predictor.

To develop an age-independent AR signature, we first identified AR associated genes in adult samples using the 20,739 probe sets whose expression was not significantly different between adult and pediatric samples. We compared allograft gene expression differences between samples with AR within 5 years after kidney transplant to samples without any rejection over five years (STA). We also determined differences in gene expression between adult and pediatric TCMR and CAN. We further identified genes whose expression patterns were significantly associated with AR-free survival using Cox-regression survival analysis. Because there was limited long-term follow-up for patients with peripheral blood expression data available in public databases, we determined differences between patients with AR and those that were stable over 2 years. As shown in **Figure 3**, these four tests, that were independently performed in either kidney tissue or peripheral blood, identified 90 probe sets whose expression were significantly associated with AR in both allograft and peripheral blood samples by either Mann-Whitney U-Test compared to STA (p<0.001) or by Cox-regression survival analysis (p<0.001) (**Table S1**). This probe set group (AR90sig) was then utilized to train and test multiple models across sample groups.

### Biologic Validity of Candidate Genes

**Figure 4:**
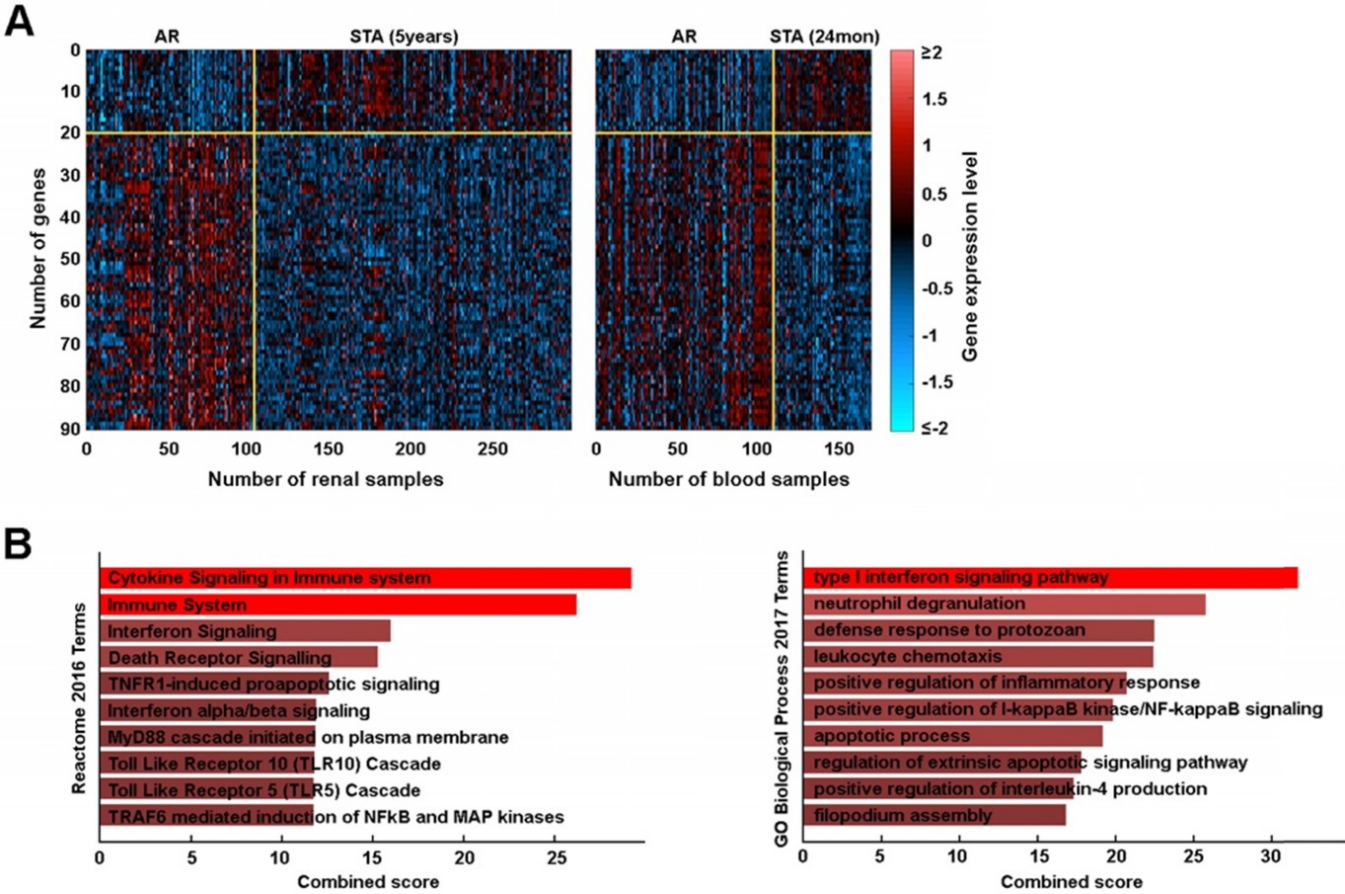
** An AR associated gene set. (A)** Heatmap of 90 probe set expressions in renal and blood training sets. **(B)** *Reactome* pathway analysis and *GO* Biological Processes.

We plotted these genes on a heatmap to define their expression between groups which confirmed good segregation between AR and STA groups (**Figure 4A**). From the 90 AR associated probe sets, we identified 76 genes whose expressions were significantly changed in AR. To determine the biologic basis of these 76 genes, we performed gene set enrichment analysis and found our gene signature was significantly associated with immune system and interferon signaling (**Figure 4B**, **Table S2**). Furthermore, we defined a novel gene network using *STRING v11* that included the pathways noted above as well as others (**Figure S1**).

### Developing a 90-probe set identifier of acute rejection

**Figure 5:**
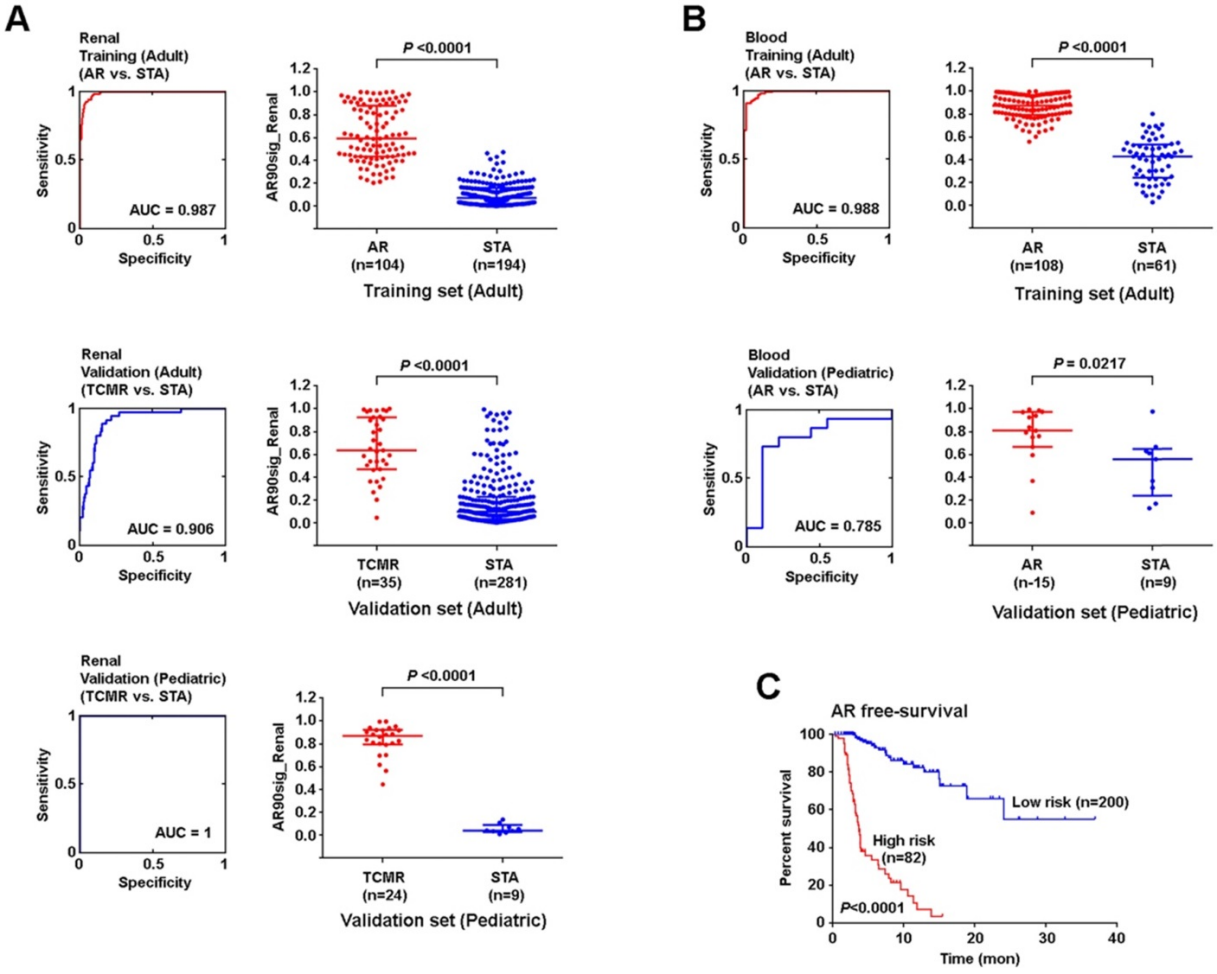
** An age-independent signature AR in renal parenchymal and peripheral blood samples. (A)** 90-probe set model for the identification of AR event 5 years post-transplant using renal tissue samples. **(B)** 90-probe sets model for the identification of AR event 5 years post-transplant using blood cells. **(C)** AR-free survival between high and low AR risk groups defined by renal AR signature. ROC curves are plotted with AUCs noted (left panel). Logistic regression analysis was performed using non-parametric Mann-Whitney U test, lines represent median and interquartile range.

Using SSS modeling, we next created a 90-probe set predictor using a training set of 298 adult kidney allograft samples and validated this in independent sets of adult (n=316) and pediatric (n=33) samples (**Figure 5A**). All three analyses showed high sensitivity and specificity for the signature to identify AR. Because the kidney tissue and peripheral blood samples were normalized differently (as they are from different tissue compartments with different background variability), we built and validated renal tissue and peripheral blood models independently. Therefore, we next created a separate signature in adult peripheral blood (n=196) samples, and validated this model using an independent set of pediatric peripheral blood (n=24) samples (**Figure 5B**). Though the two signatures contained the same genes, SSS was run on allograft and peripheral blood samples independently, yielding different coefficients. Of note, the 90-probe set signature performed well on ROC analysis with a minimum area under the curve (AUC) of 0.79 when considering both analyses.

Furthermore, we created a cut-off gene expression level at maximum sensitivity and specificity in training data to define high vs. low risk of AR and applied this cut-off to validation sets. The positive predictive value (PPV) in the adult renal validation set was 30%, while the negative predictive value (NPV) was at 98%. The model also successfully delineated AR event-free survival between high vs. low risk cases (p<0.0001, Mantel-Cox test) (**Figure 5C**). In peripheral blood, a similar analysis was performed which showed a PPV of 85% and NPV of 70% in the pediatric validation dataset (**Table S3**)**.**

### Creating an age-independent 8 gene signature of early onset AR

In order to monitor early AR events, we obtained blood samples from AR (n=42) and STA (n=47) patients available from our institutional biorepository. All samples were from patients monitored for one year post transplant, with STA defined as no rejection during that time. All AR samples were obtained within 30 days prior to an AR event. Patients were excluded from the AR group if they experienced another event (e.g. an infection) up to 14 days after the rejection event. Patients in the two groups were demographically similar except with regards to immunosuppressive management. More patients in the AR group received basiliximab induction and/or belatacept maintenance, while patients in STA group received tacrolimus (**Table 1**).

**Figure 6:**
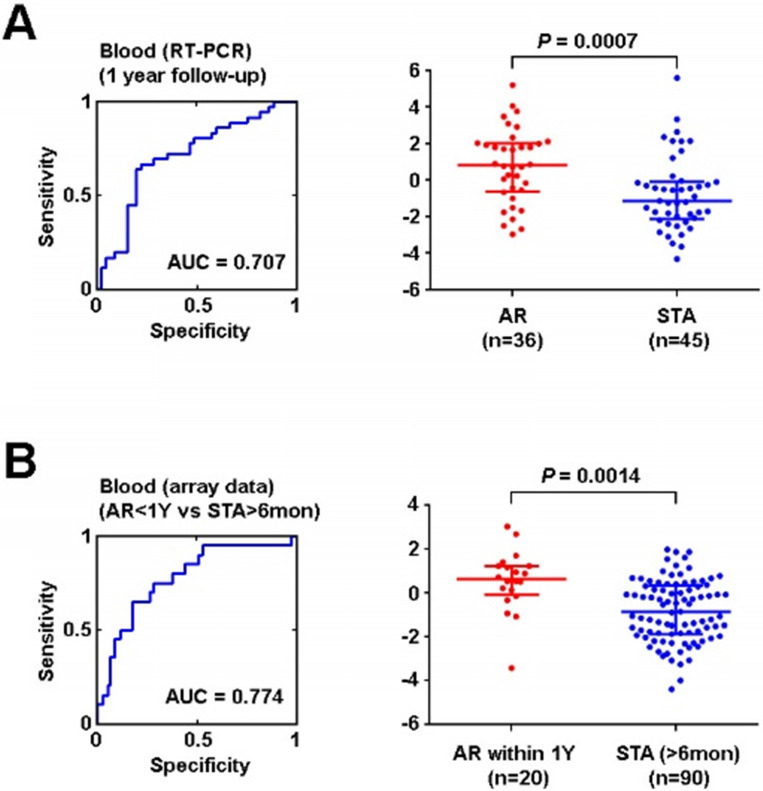
** Validation of AR signature *in vitro* and *in silico* using independent datasets. (A)** ROC curve of 8-gene signature of AR within 1 year after kidney transplant using institutional peripheral blood samples (training set) (**B**) ROC curve of 8-gene signature of AR event within 1 year after kidney transplant by *in silico* analysis (testing set). Logistic regression analysis was performed using non-parametric Mann-Whitney U test, lines represent median and interquartile range.

After removing genes located on the X chromosome and probe sets related to microRNA, a total of 76 genes corresponding to our original 90 probe sets were interrogated by Real Time-Polymerase Chain Reaction (RT-PCR). 8 genes (DIP2C, ENOSF1, FBXO21, KCTD6, PDXDC1, REXO2, HLA-E, and RAB31) were found to be differentially expressed in AR and this signature retained its significance after adjusting for multiple clinical variables, including as race, gender, age, and treatment (use of depletional induction, and/or use of belatacept based maintenance immunosuppression) (**Table S4**). Using these 8 genes, PCA was again employed to create a model to identify AR. The ROC curve AUC was found to be 0.71(**Figure 6A**). Finally, we applied this signature of early AR events to patients the microarray data that we samples in the GEO we had initially queried, including 110 patients (adult and pediatric) that either experienced AR within 1 year or were stable for at least 6 months. Utilizing the PCA we created with our institutional cohort, we applied the 8 gene signature which yielded an AUC of 0.77 in this cohort (**Figure 6B**). The NPV and PPV for the institutional dataset were 74.5% and 70.6% respectively. The NPV and PPV for the validation in the public dataset were 83.2% and 66.7% respectively (**Table S3**).

## Discussion

In the present study, we created and validated a gene signature for AR using both publicly available kidney allograft parenchymal and peripheral blood gene expression data and peripheral blood biospecimens from our institutional biorepository. After creation of a 90-probe-set signature targeting 76 genes based on microarray data, validation of our allograft biopsy signature showed a very high AUC in adult (0.91) and pediatric (1.00) datasets. In peripheral blood, our validation AUC in a pediatric cohort was moderate at 0.79. Examination of our institutional cohort identified a subset of 8 differentially expressed genes. We confirmed this 8 gene signature in a cohort of 110 patients from public databases and again demonstrated a reasonable AUC for identifying early acute rejection (0.77). Overall, our analysis demonstrates an effective method for biomarker discovery utilizing a combination of publicly available data and single center resources. We report an age-independent signature of AR that performs well in a peripheral blood assay despite diverse and non-standardized immunosuppressive regimens.

Though there is considerable excitement regarding the ability of peripheral blood-based biomarkers to advance the diagnosis and treatment of disease, there have been great challenges in moving from the research setting into clinical care 43. Additionally, all biomarker research has been plagued by a lack of reproducibility 44. Given these limitations, novel methods of merging available data in all relevant combinations to imbue richness in analysis is needed. Previous studies have utilized multiple datasets, including across transplantation disciplines, to create signatures of rejection 18. We expanded this idea further by utilizing all relevant tissue compartments. By building the base set of differentially expressed genes from both kidney allograft and peripheral blood gene expression data, we allowed for the detection of a very broad set of relevant genes involved in the AR response. Prior studies have failed to find a strict correlation between genes active in the graft and peripheral blood at the time of AR 45,46. Our current study, however, shows that it can be effective to utilize genes differentially expressed in either compartment in the determination of molecular perturbations in both.

Mechanistically, our 90-probe set signature contained 76 genes, many of which are important in immune regulation. One central pathway in our signature is that of Tumor Necrosis Factor-α (TNF-α) and the nuclear factor κ-light-chain-enhancer of activated B-Cells (NFκB) signaling. This multifaceted pathway is important in pro-inflammatory and apoptotic mechanisms depending on the context 47,48. Our signature of AR was also associated with inflammatory TNF signaling as MCL1, a known anti-apoptotic factor important in both polymorphonuclear cell and lymphocyte survival 49. Additionally, there was upregulation of USP4 and NFKBIA, both of which downregulate TNF-α based NFκB signaling. These mediators may attenuate overall TNF-α signaling to prevent exhaustion of activated cells 50. Additionally, some reports in transplantation have noted certain polymorphisms of NFKBI are associated with AR, suggesting that some forms of this gene product may enhance pro-inflammatory signaling 51. NFKBI is necessary for TNF signaling as it holds NFκB in the cytoplasm prior to nuclear translocation and activation of its inflammatory transcriptional program 48.

Consistent with our initial analysis, we saw upregulation of Human Leukocyte Antigen (HLA)-E in our subset of 8 genes that were associated AR arising within 1-year post-transplant. HLA-E interacts with CD159c/NKG2C, which activates NK cells. This HLA-E mediated signaling has been shown to occur in the kidney during AR 52. Interestingly, HLA-E upregulation has been noted as a “Universal” rejection feature of AR, regardless of histologic type 53. Additionally, two other Class-I HLA presentation associated transcripts, KCTD6 and FBX021, are implicated in our gene signature. Both are involved in ubiquitination and antigen processing, suggesting a contribution of increased antigen presentation as a contributing factor to rejection 54.

Although our study provides an age-independent gene signature that was validated in multiple pediatric and adult cohorts, our study has limitations. First, the data from which the initial signature was created are heterogenous. It is possible that there is unmeasured methodologic variance for which we cannot correct with our normalization methods. Definitions of endpoints between studies are also various, which could contribute to miscategorizations of rejection or stability. However, we favor these differences to be small and likely randomly distributed. We also note that the PPV of the validation set among adult renal tissue samples is low (31%). This may be because the differentially expressed genes with the most predictive power were identified initially in the peripheral blood datasets. Though this presents difficulty with pursuing this gene signature in biopsy tissue, it bodes favorably for continued investigation of this signature in peripheral blood. Moreover, we cautiously interpret our positive results as previous investigators have failed to corroborate gene signatures between peripheral blood and kidney biopsy tissue 24. However, we provide a much larger sample size in the present study which may account for an increased ability to detect similarities between the two compartments.

With regards to the corroboration of the microarray data with our newly generated RT-PCR data, there is a well-known and reported discordance between the two assays 55. However, we believe these difficulties only raise the threshold for identifying meaningful differences in gene regulation. Additionally, as with all models that contain numerous variables, there is the possibility of overfitting the model. Finally, the samples for our validation cohort were from an institutional biobank. Prospective validation of our assay is necessary and would be the next appropriate step in its development.

## Conclusion

Acute rejection remains a significant problem after kidney transplantation. Less invasive methods of identifying acute rejection are important to maximize graft survival and minimize patient morbidity. We identified and validated an age-independent peripheral signature of acute rejection that is effective in the setting of diverse, non-standardized immunosuppressive therapies. This was done efficiently by utilizing prior datasets to define our candidate signature before validating in an institutional cohort. The conduct of a prospective trial to further validate this signature is warranted.

## Supplementary Material

Supplementary figures and tables.

## Figures and Tables

**Table 1:** Demographics of institutional cohort

Characteristic-n(%)	Rejection- 42(39)	Stable- 47(44)	P-Value
**Age**-mean(SD)	41(17)	39(21)	0.63
**Pediatric**-n(%)	8(19)	15(32)	0.23
**Female Sex**-n(%)	17(41)	20(43)	1.0
**Race**-n(%)			0.054
African American	24(57)	13(28)	
Asian	0(0)	2(4)	
White	16(38)	30(64)	
Other	2(5)	2(4)	
**Transplant Type**-n(%)			0.162
Living Donor	13(38)	8(25)	
Deceased donor	21(62)	24(75)	
**Induction Type**-n(%)			
Basiliximab	14(33)	7(15)	0.049
Anti-Thymocyte Globulin	9(21)	9(19)	0.79
No Induction	19(46)	31(66)	0.058
**Maintenance Therapy**-n(%)			
Prednisone	40(95)	39(82)	0.095
Tacrolimus	35(83)	46(98)	0.024
Mycophenolate Mofetil	42(100)	46(98)	1.0
Cyclosporine	0(0)	1(2)	1.0
Azathioprine	1(2)	2(4)	1.0
Sirolimus	2(5)	4(9)	0.67
Belatacept	7(17)	0(0)	0.004

## Abbreviations

AR: Acute Rejection
ABMR: Antibody Mediate Rejection
AUC: Area Under the Curve
CAN: Chronic Allograft Nephropathy
GEO: Gene Expression Omnibus
IFTA: Interstitial Fibrosis/Tubular Atrophy
NFκB: Nuclear Factor κ-light-chain-enhancer of Activated B-Cells
RT PCR: Real-Time PCR
ROC: Receive Operating Characteristic
STA: Stable
TCMR: T Cell Mediated Rejection
TNF-α: Tumor Necrosis Factor-α
