# Supplementary material for: An age-independent gene signature for monitoring acute rejection in kidney transplantation
Source: Theranostics. 2020 May 25;10(15):6977–86. doi: 10.7150/thno.42110 (PMC7295062; doi:10.7150/thno.42110)
Supplement: Supplementary file 1 — Supplementary figures and tables. [file thnov10p6977s1.pdf]

For the best experience, open this PDF portfolio in Acrobat X or Adobe Reader X, or later.

Get Adobe Reader Now!
